# OD46 Inequalities In Dementia: Identifying Instruments For Measurement

**DOI:** 10.1017/S0266462324001739

**Published:** 2025-01-07

**Authors:** Sian Hodgson, Helen Hayes, Patricia Cubi-Molla, Martina Garau

## Abstract

**Introduction:**

Despite dementia being the seventh leading cause of death globally, there is relatively little discussion of the presence and impact of inequalities in this context. We explore ways to quantify the magnitude and variation over time of inequalities related to people living with dementia (PLWD) and their informal carers.

**Methods:**

We conducted a targeted literature review to identify inequalities faced by PLWD and their informal carers regarding their access to and experience of health and social care in England, Wales, and Northern Ireland. We selected four of the identified inequalities as case studies (CS) to explore data and methods that can be used to measure and monitor progress to tackle them. The CS considered were: (CS1) timely diagnosis in rural areas; (CS2) financial pressures for informal carers; (CS3) timely diagnosis in deprived areas; and (CS4) diagnosis rates for ethnic minority groups. We use data from 2018 to 2023 in England.

**Results:**

We identified 110 inequalities for PLWD and 28 inequalities for carers. For CS1, we proposed two measures: the “rurality gap” (gap in diagnosis rates between the most and least rural areas) and the “concentration index” (the extent to which diagnosis rates are distributed disproportionately between less or more rural areas). The rurality gap suggests that diagnosis rates are five to eight percent lower in rural areas in England. The concentration index supports this finding. CS2 shows that 41 percent of informal carers experience financial difficulties. Due to insufficient data, it was not possible to construct robust measures for CS3 and CS4.

**Conclusions:**

Many inequalities for PLWD and their informal carers are reported in the literature. Our CS highlight the need to improve methods and data to measure a set of inequalities, including those to calculate dementia prevalence and measure timely diagnosis. Better data is crucial now to inform value assessment of the upcoming Alzheimer’s disease treatments and avoid exacerbating existing inequalities.

